# The association between pupils´ aggressive behaviour and burnout among Danish school teachers - the role of stress and social support at work

**DOI:** 10.1186/s12889-022-12606-1

**Published:** 2022-02-15

**Authors:** Trine Nøhr Winding, Birgit Aust, Lars Peter Sønderbo Andersen

**Affiliations:** 1 Department of Occupational Medicine– University Research Clinic, Danish Ramazzini Centre, Herning Regional Hospital, Gl. Landevej 61, 7400 Herning, Denmark; 2 National Research Centre for the Working Environment, Copenhagen, Denmark

**Keywords:** Pupils´ aggressive behaviour, Burnout, Teachers, Stress, Social support

## Abstract

**Background:**

Pupils´ aggressive behaviour towards teachers is a serious problem which is slowly gaining attention and has been found to be linked to burnout. However, prospective studies investigating the role of stress and social support from colleagues and supervisor are lacking. Therefore, the aims of the present study were 1. to investigate the association between pupils´ aggressive behaviour and burnout among Danish primary and lower secondary school teachers, 2. to investigate whether the association between pupils´ aggressive behaviour and burnout depends on the level and duration of stress, and 3. to investigate whether social support from colleagues or a supervisor at the work place has a mitigating effect on the association between pupils´ aggressive behaviour and burnout among teachers.

**Methods:**

This study is a longitudinal study using data from 1198 teachers collected in two survey rounds at an interval of 1-year. Teacher-reported aggressive behaviour in pupils measured as harassment, threats, and violence towards teachers was collected at baseline. Burnout was measured at follow-up. The analyses were performed using multilevel logistic regression.

**Results:**

Statistically significant associations between harassment, threats, or violence and burnout 1 year later were found (all ORs 1.6) after adjustment for potential confounders. After further adjustment for stress, the estimates attenuated to ORs between 1.4 and 1.5, and were also statistically significant. Pupils´ aggressive behaviour in combination with low support from colleagues increased the risk of burnout, whereas the risk of burnout increased among those experiencing pupils´ aggressive behaviour in combination with receiving high support from the supervisor.

**Conclusions:**

The results indicate associations between all three types of pupils´ aggressive behaviour and burnout among teachers in Danish primary and lower secondary schools. Stress explained only a minor part of the association between teachers’ perceptions of pupils’ aggressive behaviour and burnout in teachers, and the results regarding social support were conflicting. The results of this study emphasize the growing need for preventive initiatives directed towards pupils´ aggressive behaviour, and future research should focus on exploring in depth how to support and prevent burnout in teachers exposed to aggressive behaviour.

**Supplementary Information:**

The online version contains supplementary material available at 10.1186/s12889-022-12606-1.

## Background

Pupils´ aggressive behaviour towards teachers is a serious problem that has existed for a long time but is only slowly gaining attention in the scientific literature [1, 2]. A meta-analysis investigating the prevalence of pupils´ aggressive behaviour directed towards teachers showed a pooled prevalence of 53% for any type of aggressive behaviour reported by teachers over a 2-year period [3]. Violence of a psychological nature is the most commonly reported aggressive behaviour [1, 4]. Pupils´ aggressive behaviour has been found to be linked to emotional distress, professional disengagement, discontent, and burnout among teachers [5–7]. A study by Otero-Lopez et al. showed that pupils’ disruptive behaviour and difficulty in managing conflicts are important for the level of job satisfaction and burnout in secondary education teachers [8]. Likewise, a study by Shakleton et al. showed that teachers’ perception of the school’s safety and support and pupils´ attitude toward learning were associated with burnout [9]. However, prospective studies are lacking on the association between pupils´ aggressive behaviour towards teachers and burnout. To our knowledge, only one study among teachers in Brazil investigated longitudinal effects of psychological violence by pupils or colleagues on burnout. The authors found a direct effect in the short term but not in the long term [10].

In Denmark, a representative survey among the working population shows that the frequency of pupils´ aggressive behaviour reported by school teachers, both violence and threats of violence, has increased from 13 and 16% in 2012 to approx. 20% in 2018 [11]. With regard to aggressive behaviour, participants were asked whether they had experienced threats of violence or violence at work (daily, weekly, monthly, or less frequently) during the last 12 months [12]. In Denmark, there are about 47,000 primary and lower secondary school teachers, approximately 70% of which are women and 30% men [13]. Some studies indicate that female teachers are at higher risk of burnout and are more often exposed to physical violence in the workplace [14, 15]. However, other studies find no gender difference [4] or even that male teachers are more often exposed to pupils´ aggressive behaviour [16].

It is well documented that burnout has a negative impact on teachers physical, mental and occupational health [17, 18]. The World Health Organization (WHO) considers burnout as an occupational phenomenon which arises in response to prolonged exposure to workplace stress, and defines burnout as a syndrome compromising the following three dimensions: 1. emotional exhaustion; 2. increased mental distance from one’s job, or feelings of negativism or cynicism; and 3. lack of personal accomplishment [19]. In the present study, burnout was assessed using the Copenhagen Burnout Inventory (CBI), which defines the core components of burnout as fatigue and exhaustion, and considers depersonalization as a way of coping and reduction of personal accomplishment as a potential consequence of burnout [20].

In addition to the lack of prospective information about the association between pupils´ aggressive behaviour and burnout, a more thorough understanding of the role of stress at work is needed.

Although burnout in teachers has conceptually been linked to stress and is understood to be a result of prolonged emotional stress at work [21, 22], studies have shown conflicting results regarding the association between job stress in teachers and burnout [4, 23]. Among the main sources of stress reported by teachers are administrative work, classroom teaching, relationships with colleagues, working conditions, and relationships with pupils [22]. However, few studies have investigated the role of stress in the association between pupils´ aggressive behaviour and burnout.

During recent years, there has been an increased awareness of social supports and broader cultural processes that potentially play a role in relation to the well-being and motivation of pupils at school [24], and a study by Romano et al. showed that support from both teachers and class-mates was inversely related to school burnout [25]. Moreover, previous studies have documented an association between support from colleagues or a supervisor and reduced reporting of aggressive behaviour among the working population in general [26, 27] and in teachers [28, 29]. Berlanda et al. [29] showed that social support from colleagues was associated with lower levels of harassment from pupils, and a study by Martinez et al. [28] found an association between lack of administrative support and the reporting of violence by pupils. However, we found no studies investigating the impact of social support on the association between pupils´ aggressive behaviour and burnout. As a potential modifiable work environment factor, we consider it highly important to investigate the potential mitigating effect of social support on the association between aggressive behaviour and burnout.

## Aim

The primary aim of this study was to investigate the association between pupils´ aggressive behaviour and burnout among school teachers, taking into account the role of gender, age, and seniority. A secondary aim was, to investigate whether the association depends on the level and duration of stress, and a tertiary was, to investigate whether social support from colleagues or a supervisor mitigates the association between pupils´ aggressive behaviour and burnout.

## Methods

### Design and population

**Fig. 1:**
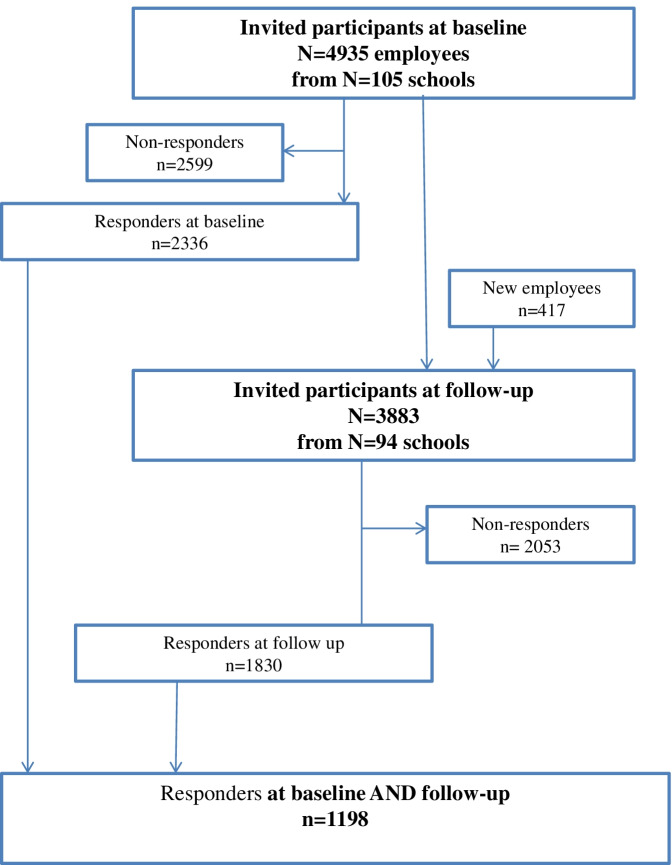
Flow chart of participants enrolled in the study

This was a longitudinal study based on two questionnaire surveys performed at an interval of 1 year. Between September and December 2018, 4935 primary and lower secondary school (grade 0-9) teachers from 105 schools in Denmark were invited to participate in the baseline survey and 2336 (47%) participated. The schools were selected randomly from a list of all municipal primary and lower secondary schools in Denmark, taking into account geographical spread and school size, so that all five main Regions in Denmark and both small and large schools were represented. The school principal gave consent for the school to participate. The teachers’ e-mail addresses were obtained from the participating schools, and questionnaires were subsequently sent to all the employed teachers by e-mail. At follow-up, 94 of the 105 schools in the baseline survey agreed to participate. In the autumn of 2019, 3883 teachers from the 94 schools were re-invited and 1830 (47%) responded. This resulted in a study population of 1198 teachers from schools of different sizes and geographical areas who responded at both survey rounds, see Fig. 1. Participation in the study was voluntary, and collected data were treated confidentially.

### Pupils´ aggressive behaviour

Information about pupils´ aggressive behaviour was based on the definition of work-related violence by Wynne, Clarkin, Cox, and Griffieths [30] and measured by questionnaires regarding harassment, threats, and violence completed by the teachers. Information was obtained from the baseline questionnaire in 2018. The respondents were asked whether they had been exposed to 10 different expressions of harassment, 7 different expressions of threats, or 12 different expressions of violence within the last 12 months.

The items about harassment concerned experiences like, e.g., being verbally patronized, having private property stolen or destroyed, or having experienced harassment by parents during the last 12 months. A total sum score with a possible range of 0-40 was calculated for each participant, and the scale was then dichotomized at the 75th percentile into high level of harassment (≥6) or low level of harassment (≤5).

Threats were measured by items asking about, e.g., being threatened with objects, written threats, or being scolded or shouted at in a threatening manner during the last 12 months. A total sum score with a possible range of 0-28 was calculated for each participant, and the scale was then dichotomized at the 75th percentile into high level of threats (≥2) or low level of threats (≤1).

Violence was measured by items asking about having experienced physically violent behaviours such as being hit, spat on, or bitten or experienced violence from pupils or parents outside work during the last 12 months. A total sum score with a possible range of 0-48 was calculated for each participant, and the scale was then dichotomized at the 75th percentile into high level of violence (≥3) or low level of violence (≤2).

Because there is no validated cut-point for the three types of aggressive behaviour, decisions on dichotomization at the 75th percentile were chosen a priori based on a desire to examine the 25% most exposed part of the sample in contrast to the rest of the sample.

### Burnout

Burnout was measured by a scale from the validated Copenhagen Burnout Inventory (CBI) [20]. In contrast to the widely used Maslach Burnout Inventory [31], the CBI focuses on measuring only fatigue and exhaustion using three rather similar scales (personal or general burnout, work-related burnout, and client-related burnout). To not overburden the participants with questions, we choose to use the personal burnout scale, which is the scale that measures whether a person experiences being exhausted due to private life, work life, or both.

The scale consists of six items (Cronbach’s alpha 0.88) measured at follow-up.

A total sum score between 0 and 24 was generated for each individual and dichotomized into ≤11, indicating no symptoms of burnout, and ≥ 12, indicating symptoms of burnout, as recommended by Borritz et al. [15].

### Stress

To measure stress in the follow-up questionnaire, we used a validated single-item question in which stress was defined as a situation where you feel tense, restless, nervous, anxious, or have difficulty sleeping at night because you are thinking about problems [32], and added an additional two questions to get a more specific measurement: 1. “Are you experiencing this kind of stress at the moment?” [32]. This information was dichotomized into low stress level (to some degree, only a little, not at all) or high stress level (very high degree, high degree). 2. “For how long have you experienced this kind of stress?”. This information was dichotomized into short stress duration (less than 2 weeks, 2-4 weeks) or long stress duration (1-3 months, more than 3 months). 3. “What do you think is causing this kind of stress? Mainly conditions at work, mainly conditions in my private life, a combination of conditions at work and in my private life, do not know/other things.”

Based on the information about stress level and duration (items 1 and 2), a new accumulated stress variable was generated with the following four categories: 1. low stress level, short stress duration, 2. low stress level, long stress duration, 3. high stress level, short stress duration, 4. high stress level, long stress duration.

### Social support

Social support from colleagues and a supervisor was measured at follow-up by the following two items from the Danish Psychosocial Questionnaire [33]: *“*Is there a sense of community and cohesion between you and your colleagues?” and “Can you talk with your immediate supervisor about difficulties you experience at work?” The items were measured on a 5-point Likert scale ranging from a very small extent (0) to a very large extent (100), and both scales were dichotomized into high support (≥75) and low support (< 75).

Finally, information about the covariates gender, age, and seniority was derived from the baseline questionnaire.

### Statistical analysis

Covariates were selected a priori based on a review of the literature. A correlation analysis between stress and burnout at follow-up was conducted, revealing a correlation coefficient (*r*) of 0.49. Except for correlations between the three main exposures and between seniority and age, none of the additional correlations exceeded *r* = 0.31 (Additional file 1). Of the 2336 responders at baseline 1138 were non-responders at follow-up (Fig. 1). A comparison between the responders and non-responders according to gender, age, and seniority was performed.

The distribution of exposure, outcome, and covariates of the study population was presented as numbers and percentile distribution. To investigate the associations between harassment, threats, or violence and burnout, multilevel logistic regression analyses, taking into account the possible correlations between teachers from the same schools, were performed. The multilevel logistic regression was based on a two-level analysis. The first level representing the 1198 teachers and the second level representing the 94 participating schools. Crude and adjusted odds ratios (OR) with 95% confidence intervals (95% CI) were calculated. Two adjusted models were performed. Model 1 adjusting for age, gender, seniority, and baseline burnout. Model 2 further adjusting for stress. Finally, the associations between the three types of pupils´ aggressive behaviour and burnout were stratified for each of the two measures of social support (from supervisors and from colleagues). All statistical analyses were performed using the statistical software package Stata, version 16.1 (Stata Corporation, College Station, Texas, USA).

### Ethical considerations

The study was approved by the Danish Data Protection Agency, which is an independent authority that supervises compliance with the rules on protection of personal data. According to Danish Law (Act on Research Ethics Review of Health Research Projects), available at: www.nvk.dk/english/act-on-research (accessed on: 8 October 2021), questionnaire- and register-based studies require neither approval by ethics or scientific committees nor written informed consent. However, the participants gave informed consent by ticking a box in the questionnaire after being informed about the purpose of the study and how their own answer could be deleted if they wanted and hereby agreed to participate in the survey.

## Results

No difference was found in the gender distribution between responders and non-responders. However, slightly more teachers in the age groups ≤21–30 and > 60 were among non-responders than among responders (max. 6% difference in the age groups) and slightly more teachers with ≤5 years and > 20 years of work experience were found among non-responders than among responders (max. 8% difference in the seniority groups). As can be seen from Table 1, 75% of the teachers were women. Most teachers were in the age group 41-60 years (65%), with a seniority between 6 and 20 years (53%). Altogether, 12% reported having been exposed to high stress levels for a period of more than 1 month, and 68% reported that the stress was caused mainly by conditions at work, while 26% reported that it was caused by a combination of conditions at work and in private life. About one-fifth (21%) reported symptoms of burnout. The mean burnout score in this sample of teachers was 10.9 (not shown in Table 1).

**Table 1:**
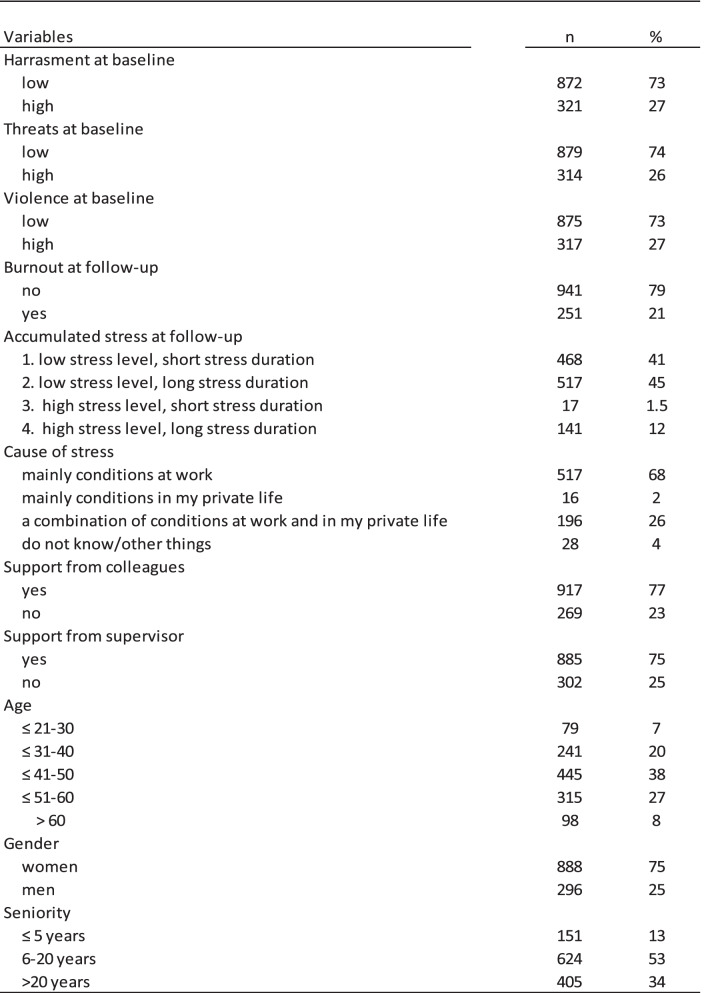
Distribution of exposure, outcome and covarites of the study population, *n* = 1198

### Pupils´ aggressive behaviour and burnout

Table 2 shows statistically significant crude associations between all three types of pupils´ aggressive behaviour and burnout 1 year later (ORs between 1.7 and 2.0). After adjusting for gender, age, seniority, and baseline burnout, all three estimates attenuated to 1.6, but were still statistically significant. When further adjustment for stress, the estimates attenuated further (OR 1.4 and 1.5), but were also statistically significant. A strong increasing trend in the crude association between stress and burnout at follow-up was seen, with high stress level and long stress duration showing the strongest association with burnout (OR > 50). Low social support from colleagues and a supervisor was statistically significantly associated with burnout (OR 1.7 and 2.5, respectively), and women had a 1.6-fold statistically significant increased odds of being burned out compared to men.

**Table 2:**
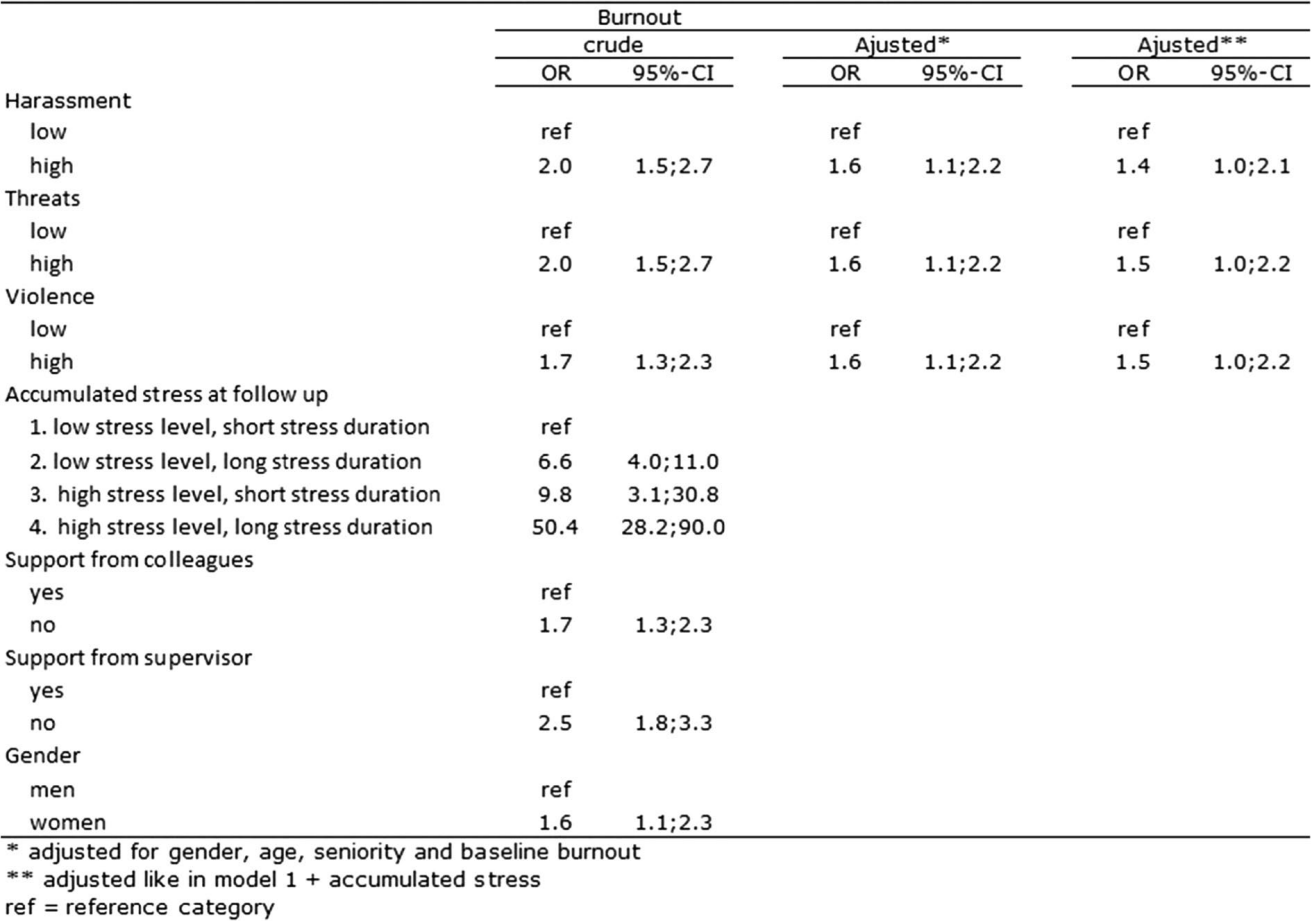
The association between harassement, threats or violence and burnout, *n* = 1198

### The impact of the level and duration of stress

Table 3 shows a stratified analysis of the association between harassment, threats, or violence and burnout among those experiencing high or low social support from colleagues.

**Table 3:**
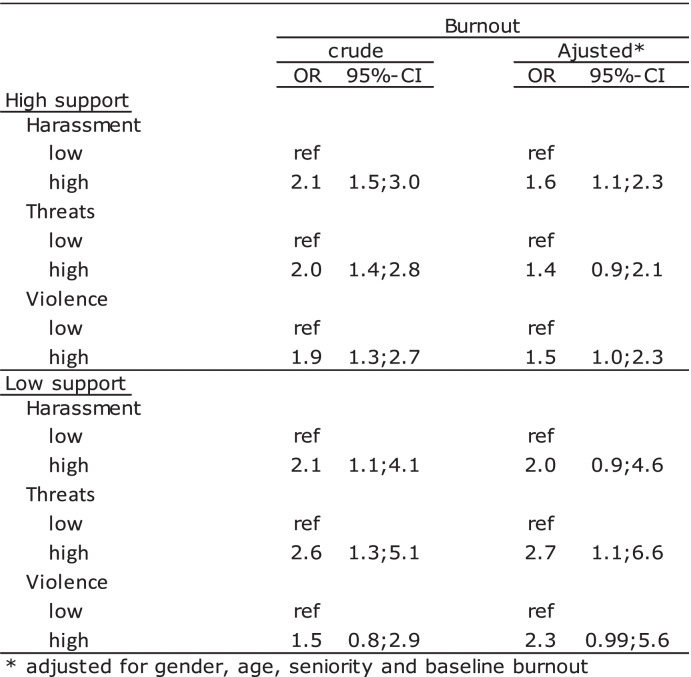
The association between harassement, threats or violence and burnout stratificed by support from colleagues, *n* = 1198

Overall, statistically significant crude associations between harassment, threats, or violence and burnout were seen both among those experiencing support from colleagues and those not, except for the association between violence and burnout among those with low support, which was statistically non-significant. After adjusting for age, gender, seniority, and baseline burnout, the associations in the group experiencing high support decreased to between 1.4 and 1.6, with harassment and violence still showing statistically significant associations. In the low support group, two ORs increased and one attenuated by 0.1, the association between threats and burnout showing the highest and only statistically significant adjusted estimate (OR 2.7, 95% CI 1.1-6.6).

### The mitigating effect of social support from colleagues or a supervisor

Table 4 shows a stratified analysis of the association between harassment, threats, or violence and burnout among those reporting high and those reporting low social support from their supervisor.

**Table 4:**
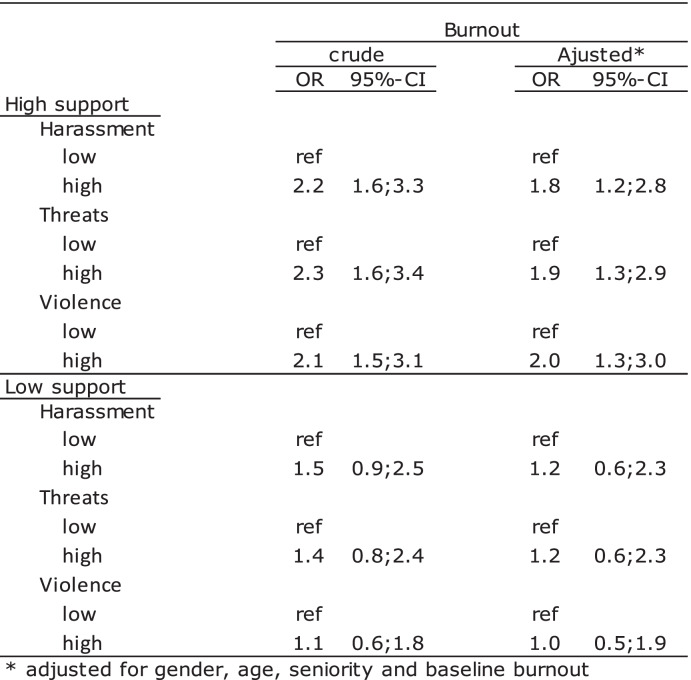
The association between harassement, threats or violence and burnout stratificed by support from supervisor, *n* = 1198

In the high support group, crude statistically significant ORs between 2.1 and 2.3 were seen. In the low support group, statistically non-significant ORs between 1.1 and 1.5 were found. After adjustment, the ORs in the high support group decreased to between 1.8 and 2.0 and were still statistically significant. Among the low support group, statistically non-significant adjusted ORs of 1.0 or 1.2 were seen.

## Discussion

The results of this study indicate an association between teachers´ perception of pupils´ aggressive behaviour towards them and burnout among school teachers when potential confounders are taken into account. The strength of the association was independent of type of aggressive behaviour. The vast majority of teachers reported that the experienced stress came from conditions at work or a combination of conditions at work and private life. Stress was strongly associated with burnout, especially among those having experienced high stress level for more than a month. However, stress only explained a minor part of the association between pupils´ aggressive behaviour and burnout. A strong association between social support from colleagues or a supervisor and decreased risk of burnout was seen. However, after stratifying the associations between the three types of pupils´ aggressive behaviour and burnout on social support, an opposite trend was seen in regard to support from colleagues and a supervisor. Pupils´ aggressive behaviour in combination with low support from colleagues increased the risk of burnout, whereas the opposite was the case for support from a supervisor, where the risk of burnout increased among those reporting pupils´ aggressive behaviour in combination with high support from a supervisor. High support from colleagues after exposure to pupils’ aggressive behaviour did not reduce the impact on risk of burnout.

With our finding of a mean burnout score of 10.9 and sum score of ≥12 (range 0 to 24) indicating symptoms of burnout, this study supports previous findings that burnout is a work environment challenge among public school teachers [6, 7, 34]. At the same time, the results show a high rate of pupils´ aggressive behaviour in the form of harassment, threats, or violence, which is consistent with previous findings [3]. Whereas previous studies in teachers have documented an association between high workload or job strain and burnout [35, 36], between interpersonal relational stress or occupational stress and burnout [35, 37] and between job demands or lack of social support and pupils´ aggressive behaviour [29], to our knowledge this is the first study to explore how teachers’ perceptions of pupils´ aggressive behaviour towards them affect burnout and in addition explore how stress and social support affect this association using a longitudinal design.

This study revealed a strong association between the reporting of stress and burnout, while only 2% of teachers reported that the stress was mainly caused by conditions in their private life. This indicates that stress conditions at work play an important role in developing burnout among teachers. However, despite the strong exposure-response association between high stress and burnout, especially if the stress level was high for a long period of time, stress only explained a minor part of the association between pupils´ aggressive behaviour and burnout, indicating the existence of other causal pathways. This means that it is not enough to pay attention to the stress levels among teachers having been exposed to aggressive behaviour from pupils, because some will be at risk of developing burnout even when not stressed.

Although low social support from colleagues in combination with experiencing aggressive behaviour from pupils increased the risk of burnout, high collegial support did not prevent burnout after being exposed to aggressive behaviour. The fact that social support from a supervisor actually increased the risk of burnout among those who had experienced aggressive behaviour from pupils was surprising. As noticed in a review by Cooper et al. [38], the results of other studies focusing on the mitigating effects of social support on different outcomes have been inconsistent. Although empirical evidence generally points to the psychological benefits of supportive relationships, received social support may be unrelated to positive outcomes, or even associated with negative outcomes [39]. An explanation for our results may be that the social support received may not be sufficient in relation to the stressors [40], in this case pupils´ aggressive behaviour. The fact that social support from the supervisors increased the risk of burnout may need to be understood in the light of the questions asked. The question about support from supervisor focuses on the availability of the supervisor to talk about difficulties at work, whereas the question asked about support from colleagues focused on emotional support and the feeling of belonging to a group. Emotional support has been found to be more healing and less controlling than informational and tangible support [41]. Another explanation for the result that social support from a supervisor did not mitigate the association between pupils´ aggressive behaviour and burnout could be that receiving support may draw more attention to the problem [40]. Receiving supervisor support may help teachers to feel safe and to share feelings about being exposed to aggressive behaviour from pupils´ [42] and consequently higher reporting of such episodes and burnout symptoms.

Major strengths of this study are the longitudinal design, the use of three different measures of pupils´ aggressive behaviour, and the use of a validated scale to measure burnout. However, the results of the present study should also be considered in the light of some potential limitations. Although 21% of the teachers in this study reported being burned out, the problem could potentially be even bigger due to the so-called healthy-worker effect [43]. One explanation could be that teachers that had been exposed to aggressive behaviour or highest stress experiences had already left the profession or the labour market. It is also possible that those with the most demanding work environment or highest stress levels chose not to participate, or are on sick-leave, and therefore are missing in the study population, which could possibly underestimate the associations studied. However, the distribution of gender and age in our population correspond to the members of the Danish Teacher Association (96% of all teachers in Denmark), and for this reason we think the risk of selection bias is limited. Likewise, a previous study using data collected from questionnaires showed that although certain characteristics were related to those who initially chose to participate and especially to those who participated at follow-ups, it did not have any large influence on the relative risk estimates measured in the studies, which is reassuring for the generalizability of the results of this study [44]. It is, however, important to state that the sample lacks homogeneity because it predominantly consists of women.

A further limitation is the use of dichotomized exposure and outcome measures, which creates a loss of information. However, dichotomizing also has some advantages because it increases the interpretation of the results. We used only the personal burnout scale from the CBI and therefore did not measure burnout (exhaustion), which participants clearly associated with work. However, the personal burnout scale measures general burnout, and we feel therefore that we measured how exhausted teachers felt in general.

The external validity and generalizability of the present study are limited to teachers in Denmark and other countries with similar welfare and educational systems, and comparison to other countries should therefore be made with caution.

## Conclusions

This study found that teacher reported aggressive behaviour in pupils increases the risk of burnout among Danish primary and lower secondary school teachers independent of type of aggressive behaviour. Although stress and social support from colleagues or a supervisor were found to be strongly associated with burnout in teachers, stress only explained a minor part of the association between pupils´ aggressive behaviour and burnout, and the results regarding social support were conflicting.

Thus, this study adds to the growing body of evidence regarding the negative impact of pupils´ aggressive behaviour towards teachers on burnout. These results emphasize the growing need for prevention of pupils´ aggressive behaviour, and future research should focus on exploring more in depth how to support teachers exposed to aggressive behaviour and thereby prevent burnout.

## Supplementary Information

**Additional file 1.** Correlation matrix of exposures and covariates.

## Data Availability

The datasets used and/or analyzed during the current study are available from the authors on reasonable request.
