# Supplementary material for: The association between pupils´ aggressive behaviour and burnout among Danish school teachers - the role of stress and social support at work
Source: BMC Public Health. 2022 Feb 15;22:316. doi: 10.1186/s12889-022-12606-1 (PMC8845238; doi:10.1186/s12889-022-12606-1)
Supplement: Supplementary file 1 — Additional file 1. Correlation matrix of exposures and covariates. [file 12889_2022_12606_MOESM1_ESM.docx]

|  | 1 | 2 | 3 | 4 | 5 | 6 | 7 | 8 | 9 |
| --- | --- | --- | --- | --- | --- | --- | --- | --- | --- |
| 1.Harrasment |  |  |  |  |  |  |  |  |  |
| 2.Threats | 0.5260 |  |  |  |  |  |  |  |  |
| 3.Violence | 0.4709 | 0.5991 |  |  |  |  |  |  |  |
| 4.Accumulated stress | 0.0721 | 0.0543 | 0.0530 |  |  |  |  |  |  |
| 5.Cause of stress | -0.1488 | -0.0662 | -0.0680 | -0.0503 |  |  |  |  |  |
| 6.Support, colleagues | -0.0100 | -0.0141 | -0.0208 | 0.0850 | -0.1217 |  |  |  |  |
| 7.Support, supervisor | 0.0963 | 0.0443 | 0.0430 | 0.1072 | -0.1039 | 0.3114 |  |  |  |
| 8.Age | -0.0705 | -0.0983 | -0.0696 | 0.029 | -0.0534 | 0.0549 | 0.1036 |  |  |
| 9.Gender | 0.0090 | 0.0292 | -0.0005 | 0.0595 | 0.0052 | -0.0060 | 0.0633 | 0.0424 |  |
| 10.Seniority | -0.1041 | -0.1323 | -0.0837 | -0.0276 | -0.0144 | 0.0336 | 0.0485 | 0.6690 | 0.0752 |

Additional file 1. Correlation matrix of exposures and covariates
